# Identification of phenolic antioxidants in Ipomoea *mauritiana jacq*. using spectrophotometric and mass spectroscopic studies

**Published:** 2014

**Authors:** Cheruthazhakkat Sulaiman, Sivadasan Pillai Geetha, Balachandran Indira

**Affiliations:** 1 *Centre for Medicinal Plants Research, Arya Vaidya Sala Kottakkal-676503, Kerala, India*

**Keywords:** *Ipomoea mauritiana*, *Phenolics*, *DPPH*, *LC-ESI-MS*

## Abstract

**Objective:** *Ipomoea mauritiana* is used in both Ayurveda and folk medicine systems. The tuberous roots are known to be diuretic, depurative, carminative, and anthelmintic. The objective of the current study was to identify phenolic antioxidants from *I. mauritiana* using spectrophotometric and LC-MS analysis.

**Materials and Methods**: An activity-guided fractionation and puriﬁcation process was used to identify the antioxidative components from *I. mauritiana *tuber. Dried mature tubers of *I. mauritiana* were extracted with 80% methanol and then partitioned by chloroform, ethyl acetate, acetone, and methanol. The acetone fraction showed the strongest 1,1-diphenyl-2-picrylhydrazyl (DPPH) radical scavenging activity among four fractions and was subjected to separation and puriﬁcation using preparative reverse-phase high-performance liquid chromatography (HPLC).

**Results**: Two compounds were separated from the acetone fraction using preparative LC fraction collector. The puriﬁed compounds were screened for their antioxidative potential using DPPH assay. The compounds were subjected to LC-MS analysis in ESI negative mode. One of the compounds was identified as Caffeoyl glucose based on the mass fragmentation.

**Conclusion:** The acetone fraction showed highest radical scavenging activity and the phytoconstituents of the same were identified by LC-MS/MS analysis.

## Introduction

*Ipomoea mauritiana* Jacq. (Syn. *I. paniculata* (L.) R. Br.; *I. digitata* Baker & Rendle) is a medicinal plant belonging to the family Convolvulaceae and is one of the source plants of ‘*Vidari*’, an Ayurvedic drug. ‘*Vidari*’ is a component of about 50 Ayurvedic formulations including Chyavanaprash. The annual industrial requirement of ‘Vidari’ is about 500–1000 Metric Tonnes (Ved and Goraya, 2008 ▶). The Ayurvedic Pharmacopoeia of India correlates ‘Vidari’ to tubers of *Pueraria tuberosa* (Roxb. ex Willd.) DC (Fabaceae) and *Kshiravidari* to *I. mauritiana* Jacq. and specify macro–microscopic characterization and chemical profiling of the raw materials for quality standardization (API, 2006 ▶). However, as per Ayurvedic descriptions both these species are attributed similar properties and are substituted by each other (Venkatasubramanian et al., 2009 ▶). Apart from tubers of *P. tuberosa* and *I. mauritiana*, tubers of *Adenia hondala* (Gaertn.) de Wilde (Passifloraceae) and the pith of *Cycas circinalis* L. (Cycadaceae) are also traded as ‘*Vidari*’ (Ved and Goraya, 2008 ▶).

 ‘*Vidari*’ is used as aphrodisiac, cardiotonic, demulcent, diuretic, refrigerant, and galactogogue (Chopra et al., 1992 ▶). The roots are sweet, cooling in action, appetizer, galactagogue, rejuvenating, stimulant, carminative, and tonic (Sivarajan and Balachandran, 1994 ▶). It is also used in emaciation, enteric fever, and spermatorrhea (Pandey, 2004 ▶). 

*I.* *mauritiana* is a much branched glabrous twining perennial shrub with large tuberous roots. The species is distributed throughout India in deciduous and evergreen forests and coastal tracts and widely naturalized in tropical parts of the world. Leaves simple, alternate, long-petioled, palmately 5–7 lobed, flowers bisexual, purple in long peduculate axillary cymes, capsule ovoid, four-celled, four-seeded with wooly seeds (Sivarajan and Balachandran, 1994 ▶; Warrier et al., 1995 ▶). It contains phytoconstituents such as taraxerol, taraxerol acetate, β-sitosterol, scopoletin, and 7-O-β-Dglycopyranosyl scopoletin (Khan et al., 2009 ▶). Distinct microscopic as well as phytochemical characters of tubers of *I. mauritiana* have been developed for quality control of crude drugs (Karthik et al., 2009 ▶). Regular intake of *I. mauritiana* tuber root powder is reported to be of beneficial use to persons suffering from or prone to coronary disease problems and diabetes (Moushumi et al., 2010 ▶).

Phenolic compounds are considered to be secondary metabolites and are derived from phenylalanine. Phenolics can be defined as substances which possess an aromatic ring and have one or more hydroxyl groups. Plants contain a large variety of phenolic derivatives, including benzoic acids, cinnamic acid derivatives, flavonoids, isoflavonoids, lignans, and tannins (Shahidi, 2000 ▶).There are about 8000 naturally occurring plant phenolics and about half of them are flavonoids. These flavonoids are closely related structures, based on the C_15_ heterocyclic nucleus of flavones and varying chiefly in the number of phenols, such as phenolics acid, phenyl propanoids, and phenolics quinones (Harborne and Boxter, 1995 ▶). It is a most widely distributed natural product in plants and over 2000 different compounds are reported occurring both in the free state and as glycosides. The major general categories are flavones, flavanones, flavanols, anthocyanidins, and isoflavones (Mukerjee, 2002).

Mass spectrometry is one of the most sensitive methods of molecular analysis and yields information on the molecular weight as well as on the structure of the analytes. Liquid chromatography-electrospray ionization mass spectrometry (LC-ESI-MS) has been recognized as a powerful analytical tool with its high sensitivity, short run time, and less use of toxic organic solvents (mobile phase) as compared with reversed phase stand-alone HPLC coupled with diode-array detector (Liu et al., 2005 ▶). LC coupled with mass spectrometry (LC-MS) is better suited for the analysis of non-volatile polar compounds in their natural form. Generation of free radicals or reactive oxygen species (ROS) during metabolism and other activities beyond the antioxidant capacity of a biological system gives rise to oxidative stress (Zheng and Wang, 2001 ▶), which plays a role in heart diseases, neuro-degenerative diseases, cancer, and ageing process (Astley, 2003 ▶).

 This concept is supported by increasing evidence indicating that oxidative damage plays a role in the development of chronic, age-related degenerative diseases, and that dietary antioxidants can oppose this, thus lower the risk of those diseases (Atoui et al., 2005 ▶). Antioxidants are substances that when present in low concentrations, compared with those of an oxidisable substrate significantly delay or prevent oxidation of that substance (Halliwell and Gutteridge, 1989 ▶).

In the present study, different fractions of *I. mauritiana* were screened for their antioxidant activity and mass spectral studies were carried out for the identification of major constituents in active fraction. 

## Materials and Methods

**Plant material**

Mature tubers of *I. mauritiana* were collected from the germplasm maintained at Centre for Medicinal Plants Research (CMPR), Arya Vaidya Sala (AVS), Kottakkal, Kerala, India and authenticated by the Plants Systematics Division of the centre. The collected plant material was then shade dried and powdered.

**Chemicals**

DPPH was purchased from Sigma Chemicals (Bangalore, India), while formic acid and acetonitrile (LC-MS grade) were obtained from Burdick & Jackson, USA. All other reagents used were of analytical grade (E Merck, India).

**Extraction**

Dried tuber powder (50 g) was extracted in 80% methanol for 48 h in a Soxhlet apparatus. After filtration, the filtrate was concentrated to dryness by rotary evaporator at 48 °C, then weighed and diluted to 200 ml with 80% methanol. The solution was washed with 30 ml petroleum ether (2×15 ml) to remove lipids. Twenty ml of absolute ethanol (2×10 ml) was used to precipitate protein from the solution. After centrifugation at 10000 rpm for 20 min, the supernatant was concentrated by rotary evaporator at 48 °C. It was then fractionated into 50 ml each of chloroform, ethyl acetate, acetone, and methanol using liquid-liquid solvent extraction techniques. 

**Estimation of total phenolic content (** **TPC** **)**

Total phenolic content was determined in all of the fractions. The assay was based on the reduction of phosphomolybdate ion of Folin-Ciocalteu reagent by the phenolate ion of the samples (Singleton et al., 1965 ▶). 

A desired amount of plant extract, distilled water, and 1 N Folin-Ciocalteu reagent was taken into a tube and mixed thoroughly. After an interval of 3 min, 2 ml of 2% sodium carbonate solution was added and the mixture was allowed to stand for 30 min with intermittent shaking. The absorbance of the mixture was measured at 750 nm using spectrophotometer. Different gallic acid standards were used for obtaining a standard curve. The total phenolic content was expressed as gallic acid equivalents (GAE) in mg/g of sample.

**DPPH radical scavenging activity**

The method described by Tepe et al. (2005) ▶ was used with minor modifications. One ml of 500 μM DPPH in methanol was mixed with equal volume of the extract solution in phosphate buffer (pH 7.4). The mixture was slightly shaken and kept in dark for 20 min. The absorbance at 517 nm was monitored in presence and absence of different concentrations of the extracts. Catechin was used as the standard. The antioxidative property of catechin is manifested particularly by its ability to inhibit and scavenge free radicals (Apea-Bah et al., 2009 ▶). The acetone fraction showed the lowest EC50 value on DPPH radical scavenging activity among four fractions. The result showed that the compounds with relatively high antioxidant activity might be contained in this fraction. Therefore, the acetone fraction was subjected to further separation and puriﬁcation. 

**HPLC analysis**

The acetone fraction was subjected to HPLC analysis using Agilent 1200 preparative high pressure liquid chromatographic system equipped with prep pump, a rheodyne injector, and diode array detector in combination with Chem32 and Chemstation software. Gradient elution was performed with water/0.05% formic acid (solvent A) and acetonitrile (solvent B) in a ratio of 40(A): 60 (B) at a constant flow rate of 1 ml/min. The major peaks obtained were collected in separate vials using Agilent fraction collector.

**LC-MS analysis**

The acetone fraction was subjected to HPLC analysis. The two major peaks resolved on HPLC were collected in vials and the same was subjected to LC-MS. LC-ESI-MS analysis was conducted on Agilent 6520 accurate mass Q-TOF LC/MS coupled with Agilent LC 1200 equipped with Extend-C18 column of 1.8 µm, 2.1×50 mm. Gradient elution was performed with water/0.05% formic acid (solvent A) and acetonitrile (solvent B) at a constant flow rate of 0.3 ml/ min. Column temperature was maintained at 30 °C. 

The MS analysis was performed using ESI in the negative mode. The conditions for mass spectrometry were: drying gas (nitrogen) flow 5 L/min; nebulizer pressure 40 psig; drying gas temperature 325 °C; capillary voltage 3000 V; fragmentor volt 125V; Oct Rf Vpp 750 V.

**Statistical analysis**

Data were presented as mean±standard deviation (SD) of three determinations. Statistical analyses were performed using a one-way analysis of variance. The EC_50 _values were calculated by linear-regression analysis. Results were calculated by employing the statistical software (COSTAT, Monterey, CA 93940, U.S.A.)

## Results

**Total phenolic content**

The total phenolic content of different fractions was determined spectrophotometrically by the Folin-Ciocalteu method. The total phenolic contents of all fractions and their DPPH radical scavenging activities were shown in Table 1. The TPC was highest in acetone fraction (8.62 mg GAE/g), whereas the lowest content was in chloroform fraction (2.63 mg GAE/g). 

**DPPH radical scavenging activity assay**

 The antioxidant activities of Compound-1, Compound-2, different fractions of *I. mauritiana*, and standard catechin were shown in Table 1. The isolated compounds exhibited considerable scavenging activity on DPPH radical and the order of their activity was as follows: chloroform fraction < ethyl acetate fraction< methanol fraction < acetone fraction < compound-1 (Caffeoyl glucose) < compound-2 < catechin. 

**HPLC Analysis**

 The most active fraction, acetone fraction, was subjected to HPLC analysis using Agilent reverse phase preparative HPLC system. Three peaks were obtained at R_t _16.08, 17.30, and 18.51 as major peaks. Out of these, two peaks with higher abundance (R_t _16.08 and 17.30) were collected in vials using Agilent preparative fraction collector. These compounds were used for the identification using LC-ESI-MS.

**LC-MS analysis**

The two compounds isolated using preparative HPLC were subjected to LC-MS analysis in ESI negative mode. The total ion chromatogram (TIC) was extracted with molecular feature extraction (MFE) using Agilent MassHunter software. The mass of Compound-1 (Vial-1) was determined as 342.19, m/z ([M-H] 341.19). On further fragmentation, it yielded two major fragments with m/z 135.07 and 179.03. Based on the previously reported fragmentation pattern (Anttonen and Karjalainen, 2006), the compound was tentatively identified as Caffeoyl glucose with molecular formula C_15_H_18_O_9_. The mass of Compound-2 (Vial-2) was calculated as 162.086, m/z ([M-H] 161.07). The fragmentation of the Compound-2 could not be achieved.

**Table 1:** Total phenolic content and radical-scavenging activity of Compound 1 and Compound 2 on DPPH assay from the tubers of *Ipomoea mauritiana*

**Fractions **	**TPC (mg GAE)**	**EC**_50_ ** ( DPPH)**
Chloroform Ethyl acetate Acetone Methanol Compound-1 Compound-2 Catechin	2.63± 0.17 6.53± 0.23 8.62± 0.33 8.2± 0.16 ---- ------ ------	33.58 ± 0.38 20.85 ± 0.5 12.16 ± 0.38 15.8 ± 0.78 3.86 ± 0.16 3.62 ± 0.20 3.40 ± 0.12

Each value was expressed as the mean±SD (n = 3).

**Figure 1:**
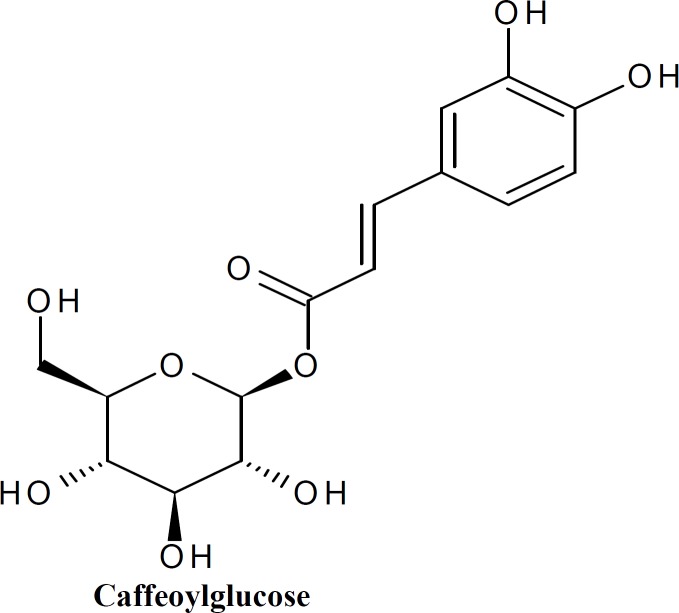
Caffeoyl glucose

**Figure 2:**
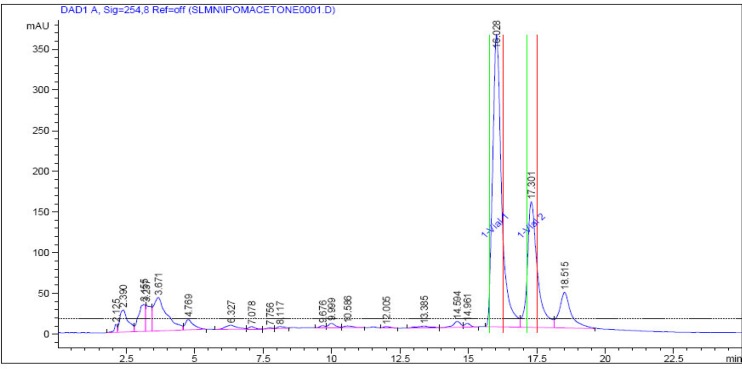
HPLC fingerprint of acetone fraction

**Figure 3:**
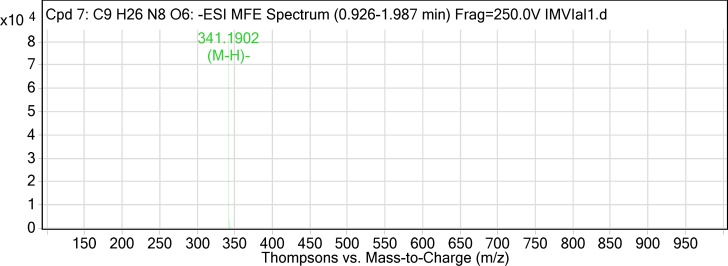
ESI-MS spectrum of Compound-1

**Figure 4:**
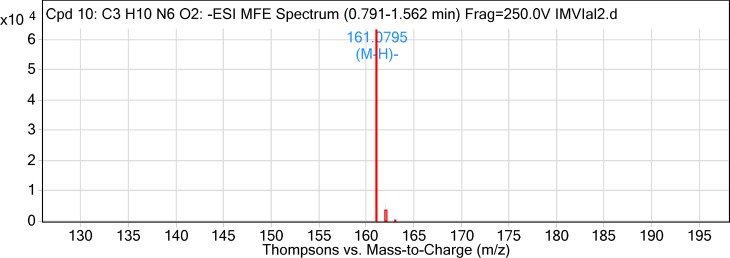
ESI-MS spectrum of Compound-2

## Discussion

The TPC was highest in acetone fraction (8.62 mg GAE/g), whereas the lowest content was in chloroform fraction (2.63 mg GAE/g). The results showed that the polarity of the solvent can affect the total phenolic content. Aqueous acetone has been shown to be a more efficient extraction solvent for hydroxycinnamic acids and anthocyanins (Heinonen et al., 1998 ▶).Free radicals can adversely alter lipids, proteins and DNA and have been implicated in aging and a number of human diseases. Lipids are highly prone to free radical damage resulting in lipid peroxidation that can lead to adverse alterations. Free radical damage to protein can result in loss of enzyme activity. DNA damage can result in mutagenesis and carcinogenesis (Sivanandham, 2011 ▶).

Among the different fractions, the acetone fraction showed lowest EC_50_ (12.16) indicating their highest scavenging activities whereas the chloroform fraction showed least scavenging activity (EC_50_ 33.58 µg/ml). The ethyl acetate, acetone, and methanol fractions showed significant radical scavenging activities. This is due to presence of high content of phenols, as polyphenols play an important role as antioxidants in living systems due to the presence of hydroxyl groups in *ortho*- and *para- *positions (Lapornik et al., 2005 ▶). 

The separated compounds showed highest antioxidant activity compared with standard catechin (EC_50_ 3.4). Compound-1(Caffeoyl glucose) showed EC_50_ 3.86 and that of Compound-2 was noted as 3.62. The radical scavenging activity of Caffeoyl derivatives has been reported previously. The present result was consistent with the report of (Gao et al., 1999 ▶). The two compounds separated using preparative HPLC possessed maximum radical scavenging activity indicates that they are the most active constituents of *I. mauritiana *against free radicals.

The acetone fraction was found as active fraction against free radicals. This may be due to the higher phenolic content of acetone fraction. The isolated compunds from the acetone fraction showed EC_50 _values 3.8 and 3.6 which are very close to that of standard catechin. The compound identifid using MS/ MS analysis supports the result. The Caffeoyl glucose belongs to the group of hydroxycinnamic acids, which has the ability for radical scavenging activity due to the presence of hydroxyl group (Herrero et al., 2009 ▶). The radical scavenging potential of different fractions of *I. mauritiana *is well established in the present studies. The therapeutic action of *I. mauritiana *may be due to its high phenolic contents. 
